# Decolonising medical education and exploring White fragility

**DOI:** 10.3399/BJGPO.2020.0147

**Published:** 2020-11-25

**Authors:** Joseph Hartland, Eva Larkai

**Affiliations:** 1 University of Bristol Medical School, Bristol, UK

**Keywords:** Inequalities, Medical humanities, Ethnic groups

## Introduction

On 17 August 2020, the BBC journalist Smitha Mundasad^1^ published a carefully researched article exploring the actions of the University of Bristol Medical School (BRMS) as it seeks to decolonise its curriculum and challenge structural forms of racism. In the subsequent days, this story was adapted and reproduced online by multiple news outlets^2,3^ who chose to preserve quotes from the only White male member of staff interviewed, exposing an important bias within the media. This article explores the subsequent abuse BRMS members experienced following these publications, why we believe this occurred, and why — despite this — we firmly stand by our commitment to confronting racism in medical education.

### Key concepts

The work taking place at BRMS focuses on two key concepts that we believe are integral to achieving a curriculum that is fair to our students and staff, and serves the needs of a modern and diverse NHS.

The first of these is ‘decolonisation’, first appearing in academic discourse in 2011 and stemming from the 1990s drive to create more inclusive curricula.^4^ Championed by students organising the ’*Why is my curriculum so* *w* *hite* *’* protest,^5^ it was predominantly driven by the humanities, and has only more recently been applied to the field of medical education. Decolonisation seeks to examine and restructure curriculums that were designed within a colonial mindset, which centralises the White, eurocentric male’s narrative above all others.^6^ A decolonisation approach to medical curriculums asks us to reflect on the ways we discuss and present race within our teaching, and how to achieve authentic representation; for example, the absence of darker skin in the teaching of clinical signs and dermatology.

The second key concept is that of ’anti-racism’. Anti-racism asks us to acknowledge that racism occurs all around us and takes various forms in our everyday life, from interactions between individuals to societal forms of structural racism that govern the opportunities a person has access to. Being anti-racist is a choice that we make every moment of every day, to challenge ourselves and the systems in which we work and teach. Within education, this may manifest as counteracting stereotypes in learning material, challenging students to consider racial bias in their clinical thinking, or reflecting on the attitudes and behaviours that students will pick up via the ‘hidden curriculum’.^7^ This is especially relevant following the events of the summer of 2020. With increased attention to the *Black Lives Matter* movement, and the concept of racism as a public health issue entering mainstream medical academic discussions,^8^ it is more important than ever that all medical schools make a conscious choice to challenge racism inherent in their curriculums and organisations.

### The controversy

A lot of the controversy following the news articles stemmed from this belief that medical curricula have racism inherently built into them. Numerous readers agreed with the importance of teaching clinical signs in darker skin, but highlighting this structural racism was where we ’lost‘ them in our argument to decolonise our teaching. Subsequently this was used as a justification to misrepresent the work, and even used to excuse online racism and aggression against the authors. Racist abuse was aimed at the Black student interviewed for the news article, and both the White lecturer and Black student’s intelligence and credentials were targeted to discredit the argument. National publications sought to bring into question the motivation for the work, claiming the authors were attempting to weaponise words to *‘* *promote a progressive agenda* *’*.^9^ Both authors experienced threats of violence.

Despite this backlash, the authors still believe that changes to images and lecture content in isolation are not sufficient to truly embed an anti-racist approach or decolonise a curriculum. We must also examine the underlying culture that has given rise to teaching that excluded the representation of Black people’s health from the mainstream narrative, inevitably perpetuating the existing health inequalities within Black, Asian, and Ethnic Minority (BAME) communities.^10^ If you doubt this ethnocentric impact on the medical consciousness, take a moment to perform an online image search for the rash associated with meningococcal meningitis. How many examples of this life-threatening clinical sign do you see demonstrated on skin that is not White? As a student, or even a parent, what message does this send?

If medical education is to ever create sustainable change, it must challenge the underlying power structures and systemic racism that has silenced these conversations until now. We urge you to consider how powerful a statement it is to exclude Black skin from medical education, and what narrative this supports.

### White fragility

The overt racism that followed the publication of the news articles was clearly indefensible, and while we cannot underestimate the impact this can have on those who experienced it, the more subtle forms of discreditation and aggression are harder to challenge and are good examples of ’White fragility’. Coined by Robin DiAngelo,^11^ this theory deals with the emotional and behavioural response that White people exhibit when challenged with their participation in, and how they benefit from, racism. DiAngelo explains how these responses are all subconsciously designed to silence conversations around race and maintain the comfortable, White status quo. The threats, discreditation, and aggression we received were designed to do exactly that: stop us discussing racism in medical education and our failure to already have a racially diverse medical curriculum. For many readers, it was acceptable for us to want to diversify our curriculum content, but by framing this within a conversation about racism we had crossed a line. However, the feeling of discomfort that elicits White fragility also means we are examining the right systems, challenging the right concepts, and asking ourselves the right questions to confront racism.

## Final thoughts

Despite the backlash following publication of these news articles, we are more committed than ever to decolonising our curriculum and strengthening our anti-racist approach. Clearly the racism that followed the publication is more than simple fragility, but the attempts to discredit the authors and belittle the work are specific acts designed to silence the very discussions we need to have if we are ever going to make a sustainable change in medical education. White fragility exists and is powerful, but if you choose to undertake this work be assured that you are not alone. We cannot allow these behaviours to hinder our drive towards long-term institutional culture change.

The time for White medical educators to stand alongside their colleagues who experience racism is long overdue. Together we must challenge racism and look for the voices lost from our curriculums. Although this article discusses the negative feedback resulting from the media attention, it is important to note this work has already inspired others and we have received fantastic feedback from the public. We leave you with this example from a Black mother who wrote to us, demonstrating why we will continue this fight, and why we encourage others to join us:

*’* *As a mother reading medical advice and it never being relevant for my daughter’s dark skin* [this] *has always been a problem and a source of worry. This is really necessary, thank you!* *’*
